# Artificial-Intelligence-Assisted Detection of Metastatic Colorectal Cancer Cells in Ascitic Fluid

**DOI:** 10.3390/cancers16051064

**Published:** 2024-03-05

**Authors:** Hyung Kyung Kim, Eunkyung Han, Jeonghyo Lee, Kwangil Yim, Jamshid Abdul-Ghafar, Kyung Jin Seo, Jang Won Seo, Gyungyub Gong, Nam Hoon Cho, Milim Kim, Chong Woo Yoo, Yosep Chong

**Affiliations:** 1 Department of Pathology, Seoul National University Bundang Hospital, Seongnam 13620, Republic of Korea; hyungkyungjkim@gmail.com (H.K.K.); hyopros@gmail.com (J.L.); 2 Department of Pathology, Samsung Medical Center, Seoul 06351, Republic of Korea; 3 Department of Pathology, Soonchunyang University Hospital Bucheon, Bucheon 14584, Republic of Korea; 4 Department of Hospital Pathology, The Catholic University of Korea College of Medicine, Seoul 06591, Republic of Korea; kangse_manse@catholic.ac.kr (K.Y.); jamshid.jalal@fmic.org.af (J.A.-G.);; 5 AI Team, MTS Company Inc., Seoul 06178, Republic of Korea; 6 Department of Pathology, Asan Medical Center, Seoul 05505, Republic of Korea; 7 Department of Pathology, Yonsei University College of Medicine, Seoul 03722, Republic of Korea; 8 Department of Pathology, National Cancer Center, Goyang 10408, Republic of Korea; cwy@ncc.re.kr

**Keywords:** ascites cytology, artificial intelligence, colorectal carcinoma, metastatic carcinoma

## Abstract

**Simple Summary:**

Ascites cytology serves as a cost-effective and noninvasive primary screening test for metastatic colorectal cancer (CRC). Diagnosing metastatic carcinoma of the peritoneum based on biopsy results is challenging, and analyzing ascitic aspiration cytology shows a limited sensitivity and specificity, along with a high variability between observers. Our study aimed to develop an artificial intelligence (AI) model that enhances pathologists’ ability to accurately diagnose metastatic CRC within ascitic fluid, improving the diagnostic accuracy, specificity, and sensitivity. This deep learning approach demonstrated a high accuracy, sensitivity, and specificity in distinguishing between malignant and benign ascites. The findings from this proposed deep learning method hold significant promise for integrating AI into clinical practice to enhance the precision of diagnosing metastatic CRC cells in ascitic fluid.

**Abstract:**

Ascites cytology is a cost-effective test for metastatic colorectal cancer (CRC) in the abdominal cavity. However, metastatic carcinoma of the peritoneum is difficult to diagnose based on biopsy findings, and ascitic aspiration cytology has a low sensitivity and specificity and a high inter-observer variability. The aim of the present study was to apply artificial intelligence (AI) to classify benign and malignant cells in ascites cytology patch images of metastatic CRC using a deep convolutional neural network. Datasets were collected from The OPEN AI Dataset Project, a nationwide cytology dataset for AI research. The numbers of patch images used for training, validation, and testing were 56,560, 7068, and 6534, respectively. We evaluated 1041 patch images of benign and metastatic CRC in the ascitic fluid to compare the performance of pathologists and an AI algorithm, and to examine whether the diagnostic accuracy of pathologists improved with the assistance of AI. This AI method showed an accuracy, a sensitivity, and a specificity of 93.74%, 87.76%, and 99.75%, respectively, for the differential diagnosis of malignant and benign ascites. The diagnostic accuracy and sensitivity of the pathologist with the assistance of the proposed AI method increased from 86.8% to 90.5% and from 73.3% to 79.3%, respectively. The proposed deep learning method may assist pathologists with different levels of experience in diagnosing metastatic CRC cells of ascites.

## 1. Introduction

Ascites cytology is a cost-effective, noninvasive primary screening test for metastatic colorectal cancer (CRC) in the abdominal cavity. CRC is the second most common cancer worldwide and has a high mortality rate. At diagnosis, synchronous peritoneal metastases account for approximately 4–17% of CRC cases, and metachronous metastases account for 25–50% of cases [1,2,3]. However, metastatic carcinoma of the peritoneum is difficult to diagnose based on biopsy findings, and ascitic aspiration cytology has a low sensitivity and specificity at approximately 50–70% and a high inter-observer variability. Furthermore, the examination of cytology slides necessitates the observation of individual cells, sheets, and clusters scattered on the slide, one by one. Obtaining a diagnosis using this process is labor-intensive and time-consuming, and it demands a high level of concentration. Additionally, the number of specimens available for cytological examination in Korea is increasing annually.

Artificial intelligence (AI) has been studied in many areas of medical science, showing promising results [4,5,6,7,8]. Recently, attempts have been made to overcome these shortcomings by introducing artificial intelligence (AI) for cytological examinations [9,10,11]. The first study to introduce a diagnostic-aid computer program using artificial neural networks for cytology was published in 2011. This study targeted breast aspiration cells. Subsequently, studies have applied it to cytologic examinations of cells from the thyroid gland [12,13,14,15,16,17,18,19,20,21], lungs, ovaries, pancreas, urine [9,22,23,24,25], and pleural fluid [16]. These studies were limited in their applicability to real-world diagnoses by factors such as not using digital scanned slides, focusing only on the diagnosis of a single cell, utilizing a limited number of samples, or encountering constraints in evaluating the quality of the samples. On top of this, the AI-assisted cytologic evaluation of ascites in other primary organs, including CRC, has not been reported [26].

In the present study, we aimed to develop an AI model using digitally scanned slides collected from various institutions, which underwent a quality assessment, to enhance the diagnostic accuracy, specificity, and sensitivity of pathologists in diagnosing metastatic ascitic fluid CRC. In addition, we tried to verify the performance by performing learning, verification, and external validity evaluations using a national-level dataset.

## 2. Materials and Methods

“The OPEN AI Dataset” was used to obtain the data for AI model development and testing. “The OPEN AI Dataset Project” aims to build a nationwide open dataset for the development of artificial intelligence models in cytopathology in Korea. This dataset contains 5506 non-gynecological cytology slides collected from 214 pathology laboratories across South Korea, including university hospitals, general hospitals, and commercial laboratories. The participating institutions from which data were collected for this study are shown in Supplementary Table S1. For this study, a total of 581 CRC cases were included after reviewing the slide and image quality from 24 institutions. The board-certified cytopathologists assessed the quality of the slides and determined their suitability for inclusion in The OPEN AI Dataset. Each positive cytology slide could only be submitted if it was accompanied by a corresponding biopsy. The negative controls were those extracted from non-malignant ascites. Cases with the possibility of multiple cancer types or peritoneal carcinomatosis due to other malignancies were excluded. Upon submission, the collected slides underwent primary verification by the institution’s pathologist and a secondary inspection by ten cytopathology specialists from the quality control committee. The submitted cell slides underwent checks not only for their diagnosis, but also for external quality control factors such as their cellularity, cell distribution, and staining; the cover slide location; and the presence of air and foreign substances. However, as we collected the dataset, we excluded the samples that were not eligible for AI training, such as those with a bad image quality. The collected slides were converted into digital images using various digital scanners, refined, labeled, and stored (Supplementary Figure S1).

### 2.1. Image Processing

The cells on cytology slides were distributed at different thicknesses for each slide or area within the slide, as opposed to tissue slides, which were cut to a thickness of 3–5 µm. In addition, because the cells existed as cell clusters with a three-dimensional structure, obtaining the proper cell shape was impossible by scanning a digital image with one focus. Therefore, we used the extended z-stacking image method, which merges the images formed at different levels of focus into a single image, where more precise cellular details can be identified. Conventional slides were scanned using five stack images, while liquid-based slides were scanned using one to three stack images and created as a single integrated image (Supplementary Figure S2). A whole-slide image (WSI) was created using digital scanners from 3DHistech (Budapest, Hungary), Leica (Wetzlar, Germany), and Hamamatsu (Hamamatsu, Japan).

**Figure 1:**
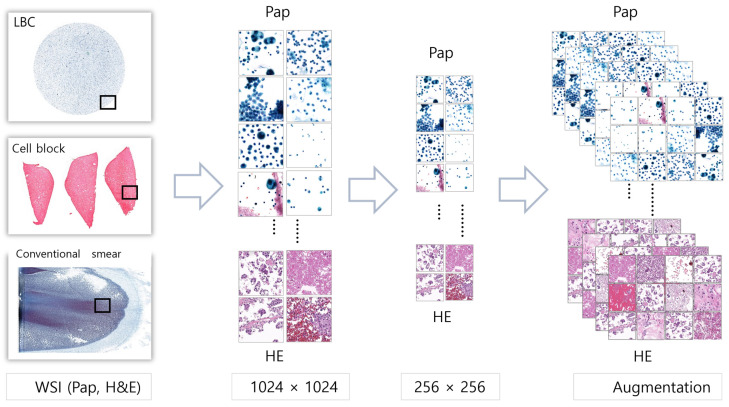
Image processing using WSIs to patch images with augmentation. WSIs were cropped into patch images with a size of 256 × 256 pixels. To enrich the dataset, these patch images were subjected to data augmentation techniques, including random horizontal and vertical flipping, as well as color corrections.

Because the size of the WSIs was too large for them to be input into the neural network directly, patch images with a size of 1024 × 1024 pixels were first cropped. The obtained patch images were then manipulated into an image with a size of 256 × 256 pixels. To increase the generalizability of the model by generating various images, the data were augmented using random horizontal flips, arbitrary vertical flips, and color corrections (Figure 1).

### 2.2. Data Distributions

A total of 392 Papanicolaou (PAP)-stained and 189 hematoxylin and eosin (H&E)-stained WSIs were included in the AI model development. We obtained 27,049 patch images of PAP-stained slides and 43,113 images of HE-stained slides. The generated patch images were randomized and stored to ensure that they did not contain personal or hospital information. Ten cytopathologists determined a diagnosis and performed a quality evaluation for each patch image. Patch images with discordant diagnoses among cytopathologists were excluded from the analysis. These image patches were randomly assigned in a ratio of 8:1:1 to the training, validation, and test sets. In the training set, 21,656 patch images from 314 PAP-stained WSIs and 34,904 patch images from 151 HE-stained WSIs were included. Table 1 summarizes the numbers of positive or negative PAP-stained WSIs with image patches and HE-stained WSIs with image patches assigned to the validation and test sets. Although the dataset for the study involved multiple centers, the AI model was tested exclusively using single-center data from the National Cancer Center (NCC).

### 2.3. Deep Convolutional Neural Networking (DCNN) Model Training

**Figure 2:**
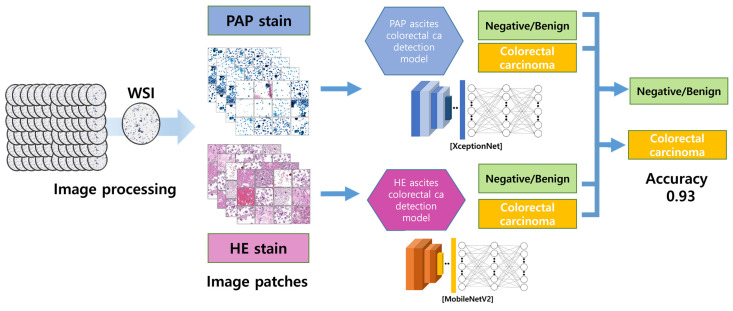
The image-processing and AI-detection-model algorithm. The DCNN models were separately trained on H&E- and PAP-stained images using the generated patch images. Xception and MobileNetV2, which had the highest binary classification accuracy, were selected as the optimal models.

DCNN algorithm models were trained to find the model with the highest binary classification accuracy for patch images. The DCNN algorithm models used were densenet161, DPN, inceptionresnetv2, inceptionv4, mobilenetv2, resnet152, resnext, senet154, Xception, and densenet161. The H&E and PAP images were trained separately, and the sensitivity, specificity, and accuracy of all models were compared (Figure 2). The AI diagnoses were generated using mobilenetV2 1.0 and Xception V1.

### 2.4. Random Patch Image Comparison between Pathologists and the AI Model

To compare the accuracy of the AI diagnostic model with that of a pathologist, four pathologists participated. One resident and three specialists with various levels of experience obtained diagnoses from image patches. The pathologists examined 1041 image patches that were not used to develop the AI models. These image patches included 845 PAP-stained and 196 H&E-stained images. The diagnoses were obtained independently, regardless of space and time. The AI model was used to diagnose the same image patches. Then, the pathologists obtained diagnoses again based on the image patches and diagnostic results of the AI model.

### 2.5. Statistical Analysis

To analyze the diagnostic agreement among pathologists, the Fleiss’ kappa coefficient was used. The analysis was performed using R statistical programming (version 3.4.1; http://www.r-project.org, accessed on 15 February 2024).

## 3. Results

### 3.1. Pretest for AI Model Selection

The AI algorithm trained on PAP-stained image patches showed sensitivities ranging from 0.553 (Desnet) to 0.7890 (Xception). In all the models except inceptioneresnetv2 (0.9992), the specificity was as high as 1.0. The sensitivity of the H&E models ranged from 0.8863 (inceptionv4) to 0.9418 (dpn), and the specificity ranged from 0.8917 (inceptionresnetv2) to 0.9973 (senet154). The accuracy ranged from 0.7748 to 0.8954 for the PAP model and from 0.8969 to 0.9667 for the H&E models. The Xception model, with an accuracy of 0.8954 for the PAP images, and Mobilnetv2, with an accuracy of 0.9667 for the H&E images, were adopted as the most accurate models (Supplementary Table S2).

### 3.2. AI Model Results

During the training phase, both the PAP and H&E models demonstrated an accuracy of 1.0000. In the validation set of the PAP model, the accuracy was 0.9424, with a sensitivity of 0.8973 and a specificity of 0.9874. Subsequently, the PAP model’s test set exhibited an accuracy of 0.8954, a sensitivity of 0.7890, and a specificity of 1.000.

In the H&E model, the validation set revealed an accuracy of 0.9603, a sensitivity of 0.9208, and a specificity of 1.0000. Meanwhile, the H&E model’s test set showed an accuracy of 0.8954, a sensitivity of 0.7890, and a specificity of 1.000.

Turning to the combined PAP and HE model, the validation set scored an accuracy of 0.9535, a sensitivity of 0.9117, and a specificity of 0.9952. In the subsequent test set, the model demonstrated an accuracy of 0.9374, a sensitivity of 0.8776, and a specificity of 0.9975. Table 2 shows the results of the H&E and PAP models.

### 3.3. Random Patch Image Comparison between Pathologists and the AI Model

Four pathologists with different levels of experience obtained diagnoses based on 1 041 patch images. Table 3 summarizes the results. The diagnostic sensitivity of the pathologists ranged from 53.9% to 91.7%, with an average of 73.3 ± 0.16%. The specificity ranged from 84.0% to 99.8%, with an average of 93.7 ± 0.07%. The accuracy ranged from 79.6% to 91.5%, with an average of 86.8 ± 0.05%. When the proposed AI model was tested on 1041 patch images, its accuracy was 86.2%. The sensitivity and specificity of the AI test were 68.6% and 97.5%, respectively. Table 3 summarizes the diagnostic accuracy of each pathologist and AI model. The pathologists obtained a re-diagnosis by referring to the diagnosis of the AI model for each patch image, and the diagnostic accuracy increased from 86.8 ± 0.05%. to 90.5 ± 0.02% (Supplementary Figure S3).

The sensitivity and specificity increased to 79.3 ± 0.08% and 94.8 ± 0.03%, respectively. In the analysis of agreement among pathologists, Fleiss’ kappa yielded a value of 0.55. Our findings indicated a moderate level of agreement among the pathologists.

### 3.4. AI-Diagnosed Patch Images

**Figure 3:**
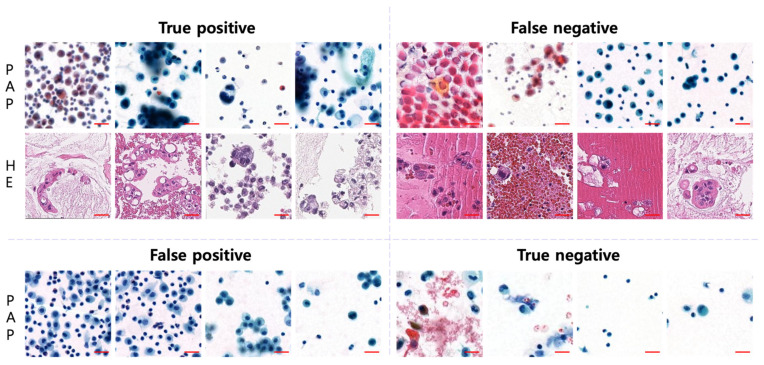
Examples of AI-diagnosed patch images. AI made incorrect diagnoses in cases with intense staining or dry artifacts, various materials in the background, or small nuclear clusters (patch image size, 256 × 256 pixels; scale bar = 50 um).

The AI-diagnosed image patches were divided into true-positive, true-negative, false-positive, and false-negative groups and compared. Figure 3 shows examples of the images corresponding to each group. The false-negative group, which was diagnosed as negative by AI despite being malignant, comprised images that were difficult to identify because of dry artifacts or strong staining. The H&E-stained images in the false-negative group contained abundant proteinaceous material and red blood cells in the background. In the PAP-stained images, a malignancy was determined if the cellularity was relatively high or if the cluster contained a few cells.

### 3.5. Disagreement between Pathologists and AI

**Figure 4:**
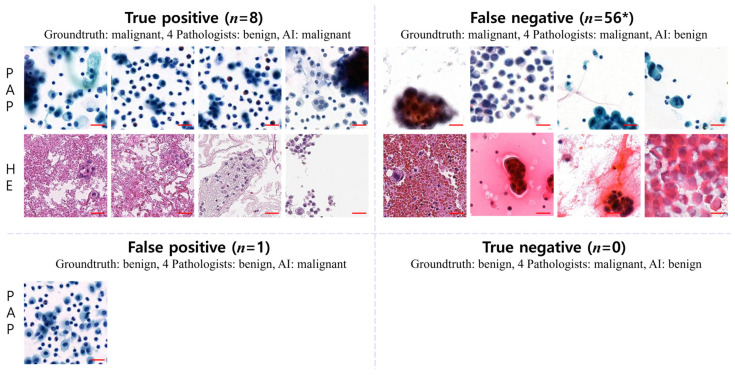
Disagreements in diagnoses between pathologists and AI. A comparison between AI diagnoses and those of pathologists revealed discrepancies. In 8 cases (true positive), all pathologists diagnosed malignant patches as benign, while only AI identified them as malignant. Conversely, no benign patches were diagnosed as malignant by all pathologists, but AI classified them as benign (true negative). Additionally, in one case (false positive, representative patches), AI interpreted a benign patch as malignant. Furthermore, 56 patches were classified as benign by AI alone (false negative) (patch image size, 256 × 256-pixels; scale bar = 50 um); * indicates representative sample patch images of false-negative cases.

When comparing the AI diagnoses with those of pathologists with various levels of experience, we observed cases where the AI results differed from the consistent results of all the pathologists. Despite the actual diagnosis being a malignant patch, all the pathologists diagnosed it as benign, while only the AI interpreted it as malignant in a total of eight cases (true positive). Conversely, in instances where the patch was benign, there were no cases where all the pathologists diagnosed it as malignant, with only the AI interpreting it as benign (true negative). Additionally, there was one patch where the actual diagnosis was benign, but only the AI interpreted it as malignant (false positive). A total of 56 patches were identified where the AI alone diagnosed them as benign (false negative) (Figure 4).

## 4. Discussion

In the present study, we successfully developed an AI classification model for differentiating metastatic CRC in ascitic fluid cytology with a high accuracy of 93%, validated its performance, and found that AI can help enhance the performance of pathologists. AI demonstrated its value in assisting pathologists, thereby elevating the overall diagnostic precision and mitigating the workload of pathologists. The collaborative approach between AI and pathologists offers a promising screening method that not only enhances accuracy, but also contributes to the efficiency of diagnostic processes. This underscores the potential of AI as an invaluable tool in pathology, serving as a robust screening mechanism that complements the expertise of medical professionals.

In 2011, a pioneering study was conducted to investigate the feasibility of using artificial neural networks in cytology, with focus on breast aspiration cells. Since then, several studies have utilized AI to examine cytological samples from various organs, such as the thyroid [4,14,16,27], lungs, and pancreas, as well as urine [9,22,23,24,25] and pleural effusion [16]. However, to date, no reports have been published on the application of AI for the cytological evaluation of ascites in the gastrointestinal or hepatobiliary tracts. This study contributes to the literature by being the first study to use AI for the cytological examination of metastatic CRC in ascitic fluid.

In 2012, Adarsh et al. presented the first instance of binary classification in effusion cytology to distinguish between metastatic carcinoma and benign cells [28]. Their study included 114 cases of adenocarcinoma, squamous cell carcinoma, and signet ring cell carcinoma. Instead of utilizing digitally scanned images, they employed a digital camera attached to a microscope to capture images and trained 30–50 cells per case. Despite being the first attempt, the origin of the effusion fluid was uncertain. Further, the number of cases was limited, and small subsets of various types of cancers were included. This study relied on spot-image photographs and differed from contemporary research, which utilizes artificial neural networks with digitally scanned pathologic slide images.

In 2020, Su et al. developed an AI algorithm to distinguish between malignant and benign cells in 487 cropped cytology images of ascitic fluid containing metastasized gastric cancer cells [27]. The algorithm achieved an accuracy of 96.80% and an AUC of 0.8851. This study evaluated individual cell images within each patch image instead of using patch images as the unit of analysis. In addition, this study had some limitations, such as the exclusion of cell clusters from the image analysis and the use of cell images obtained from a single hospital [4].

The two aforementioned studies were conducted using images obtained from a single hospital with a digital camera mounted on a microscope or by distinguishing and analyzing individual cells in the images. However, the present study was different in that it analyzed patch images obtained from digital scan slides used in actual clinical settings and predicted the diagnosis from each patch image. In addition to the two studies on ascites, previous studies on cytopathology share these common features.

Nishant et al. recently mentioned various limitations commonly encountered in non-gynecological cytology studies utilizing AI [4]. These limitations include small sample sizes, difficulties in annotation, a limited availability of z-stacked images, and a lack of well-annotated larger datasets. In addition, restricted publicly available datasets, variations in dataset annotation, and image quality were identified as limitations. Overcoming these limitations is crucial for enhancing the effectiveness and applicability of AI in non-gynecological cytology studies [4].

The most important step in developing an AI model for cytological diagnoses is the accurate annotation of the patch images used to train the AI algorithm. Even if a patch image is extracted from a WSI that is diagnosed as malignant, some patch images may contain only benign cells. Therefore, to define the ground truth value that indicates whether each patch image indicates a malignant or benign condition, exact annotation is a crucial process for creating a diagnostic AI algorithm. We ensured accuracy in this process because we used data from The OPEN AI Dataset supervised by ten cytopathologists [29,30]. The OPEN AI Dataset is a dataset created by the Korean Society of Pathology to develop AI for pathologic diagnoses. Cell slides collected for a nationwide quality control program were used for cytological data. Over 200 institutions have implemented quality control programs nationwide, including university hospitals, general hospitals, and commercial laboratories. Only patients with confirmed histological diagnoses were included in the present study. The initial diagnosis by a pathologist was reviewed at the time of the quality control submission. Quality control committee members reviewed the collected cases of cytology and tissue slides. For the cell slides collected in this manner, the committee members of the dataset were collected to evaluate the quality before and after scanning. Subsequently, image preprocessing, including image patch extraction, was performed. Ten pathologists performed image annotations and cross-quality checks (Supplementary Figure S1). This study overcomes the limitations of the previous studies by utilizing well-annotated public data of various qualities collected from diverse institutions. Additionally, most prior studies focused on PAP-stained images by utilizing liquid-based cytology [15,18], whereas this study also included H&E-stained slides, conventional smears, and cell blocks. Compared to liquid-based techniques that use membranes or filters to smear cells in a single layer, conventional smears exhibit a broader phase contrast and are associated with factors that can complicate the diagnosis, such as thick cell clusters or the deposition of proteins or mucous materials in the background. To address this issue, we synthesized z-stacked images by compositing images from three levels of liquid-based cytology and five levels of conventional smears. This study demonstrated, for the first time, the possibility of applying AI despite the limitations of conventional smears.

The sensitivity and specificity of a pathologist for ascitic fluid diagnoses are lower than those for tissue diagnoses, with significant inter- and intra-observer variabilities. This study also confirmed a significant inter-observer variability, with the sensitivity of the pathologist’s diagnosis from 1041 patch images ranging from 53.9% to 91.7% and the specificity ranging from 84.0% to 99.8%. In addition, the inter-observer variability was significant. The sensitivity of the AI diagnosis was lower than that of the pathologists, at 68.5%; however, the specificity was slightly higher, at 97.5%, and the accuracy was slightly lower, at 86.2%. The sensitivity, specificity, and accuracy improved when the pathologists obtained a re-diagnosis based on the results of the AI diagnosis. The sensitivity, specificity, and accuracy of individual pathologists depended on their experience.

How the AI arrived at these readings is unknown, as the process was black-box-like. However, analyzing false-positive and false-negative images suggested that inaccurate diagnoses may occur when the background contains substances such as red blood cells, proteinaceous material, or mucin. Additionally, limitations in detailed diagnoses of nuclear and cytoplasmic features due to factors such as a high cellularity, excessive staining, or blurriness can contribute to inaccuracies.

In reviewing cases where the AI diagnoses differed from those of all pathologists, it was found that, when malignant cells formed three-dimensional clusters that prevented nuclear characteristics from being discerned, the pathologists consistently diagnosed them as malignant, whereas AI classified them as benign, resulting in discrepancies (false negatives). Conversely, instances occurred where all the pathologists identified malignant images as benign when the malignant cells were small in size with a low nuclear cytoplasmic ratio or were sporadically distributed as single cells (true positives). Although there was one instance where AI misinterpreted a benign image as malignant, the reasons for this discrepancy were challenging to elucidate (false positive). Notably, there were no instances where all the pathologists misdiagnosed benign images as malignant; thus, no discrepancies between the AI and pathologist interpretations were found in this case (true positives).

With advances in neural network technology and digital pathology [29,30], cell pathology applications have shifted from analyzing still images obtained using digital cameras to using scanned digital images. The reading units have evolved from single cells to patch images. However, the diagnostic unit in clinical practice is the WSI. To apply this patch-level diagnosis algorithm to WSI-level diagnostic predictions, another type of algorithm is required to calculate the malignancy probability of a WSI based on the diagnostic values of the patch images. However, analyzing this process takes a long time and does not help improve the speed of obtaining diagnoses by pathologists in clinical settings. Additionally, if the ratio of malignancies among the patch images that make up the WSI is low, the accuracy of the diagnosis may decrease. Therefore, slide-level diagnostic algorithms should be developed to facilitate their use in clinical settings.

## 5. Conclusions

The present study reports the first successful development of an AI model that exhibits excellent performance in distinguishing malignant from benign image patches in ascites cytology slides of metastatic colorectal carcinoma. We utilized a dataset collected from multiple nationwide institutions comprising various slide types, stain colors, and scanning techniques, including multiple z-stacking. The AI model demonstrated a high accuracy rate of 93% and improved the diagnostic accuracy of human pathologists when they referred to the AI. This study presents an AI technique for binary classification at the patch-image level. As a result, a limitation of this study is that the predictive performance of the AI model for diagnosing WSIs cannot be guaranteed. Our findings have significant implications for the potential integration of AI in clinical practice to improve the accuracy of the diagnosis of metastatic CRC cells in ascitic fluid.

## Figures and Tables

**Table 1:** Distribution of datasets for AI model training, validation, and testing (number of whole slides/patches).

		Training	Validation	Testing	Total
PAP	Negative	281 (10,828)	35 (1353)	35 (1355)	351 (13,536)
	Positive	33 (10,828)	4 (1353)	4 (1332)	41 (13,513)
	Total	314 (21,656)	49 (2706)	39 (2687)	392 (27,049)
H&E	Negative	120 (17,452)	15 (2181)	15 (1903)	150 (21,536)
	Positive	31 (17,452)	4 (2181)	4 (1944)	39 (21,577)
	Total	151 (34,904)	19 (4362)	19 (3847)	189 (43,113)

**Table 2:** Accuracy confusion matrix of AI models.

	PAP (xceptionNet)			H&E (mobilenetv2)		
	Accuracy	Sensitivity	Specificity	Accuracy	Sensitivity	Specificity
Training	1.0000	-	-	1.0000	-	-
Validation	0.9424	0.8973	0.9874	0.9603	0.9207	1.0000
Test	0.8954	0.7890	1.0000	0.9667	0.9383	0.9958
	PAP and HE					
	Accuracy	Sensitivity	Specificity			
Training	-	-	-			
Validation	0.9535	0.9117	0.9952			
Test	0.9374	0.8776	0.9975			

**Table 3:** Comparison between pathologists and AI models in random patch image tests.

	Pathologist					Pathologist with AI	AI
	A	B	C	D	Average		
Sensitivity	70.5%	53.9%	77.1%	91.7%	73.3%	79.3%	68.6%
Specificity	96.3%	99.8%	94.7%	84.0%	93.7%	94.8%	97.5%
Accuracy	86.6%	79.6%	89.3%	91.5%	86.8%	90.5%	86.2%

## Data Availability

The data presented in this study are available from the corresponding author upon request.

## Supplementary Materials

The following supporting information can be downloaded at: https://www.mdpi.com/article/10.3390/cancers16051064/s1, Figure S1: The OPEN AI Dataset Project; Figure S2: Extended z-stacking image methods; Figure S3: Comparison between pathologists and the AI model; Table S1: Institutions participated in data collection for this study of the Open AI dataset; Table S2: Accuracy of PAP and H&E AI algorithms.
