# Supplementary material for: Artificial-Intelligence-Assisted Detection of Metastatic Colorectal Cancer Cells in Ascitic Fluid
Source: Cancers (Basel). 2024 Mar 5;16(5):1064. doi: 10.3390/cancers16051064 (PMC10931097; doi:10.3390/cancers16051064)
Supplement: Supplementary file 1 [file cancers-16-01064-s001.zip › cancers-2772185-supplementary.pdf]

Supplement Figures:

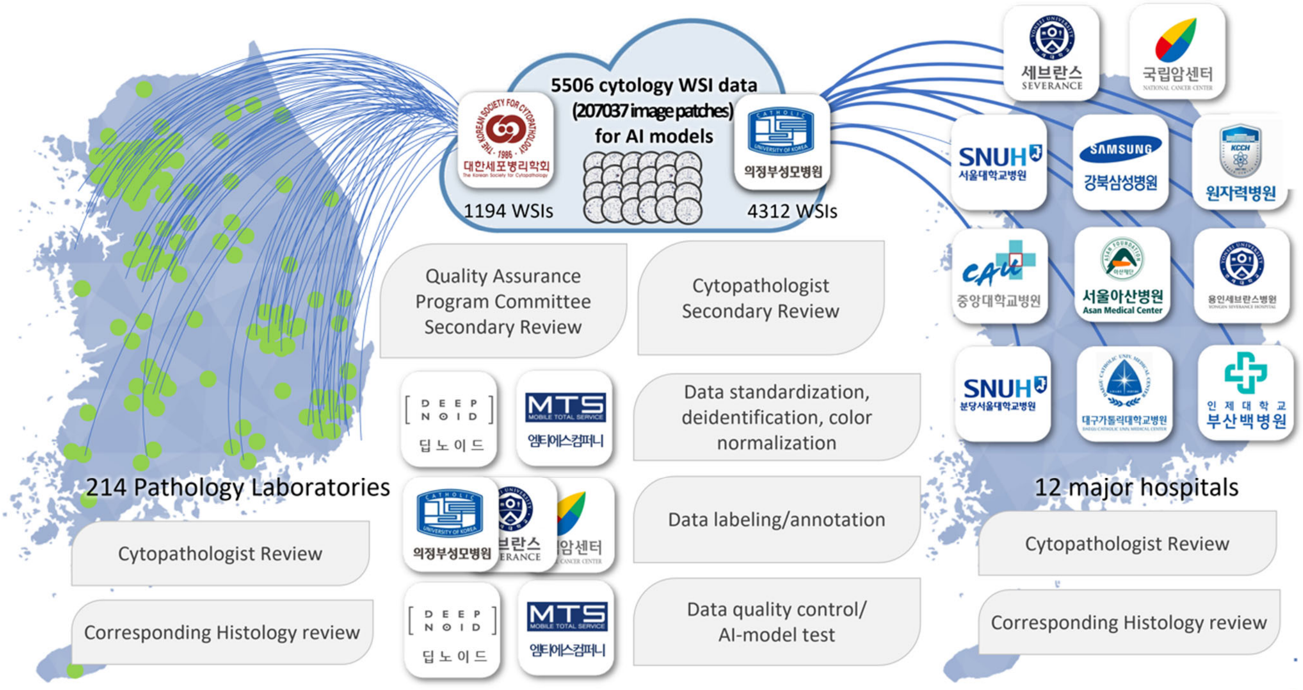

Figure S1. The OPEN AI Dataset Project.

The OPEN AI Dataset Project aims to create a nationwide open dataset for the development of artificial intelligence models in the field of cytopathology. The dataset includes non-gynecological and cytopathology slides collected from 214 pathology laboratories throughout Korea, including university hospitals, general hospitals, and commercial laboratories. Each cytology slide is collected for quality control by the Korean Society of Cytopathology with a tissue slide. Initially, each institution's pathologists reviewed the collected slides, which were then reviewed by at least 10 cytopathologists in the quality control committee. The slides were converted into digital images using various digital scanners. Subsequently, patch images were generated, and each patch image was reviewed, refined, and labeled by cytopathologists before being saved.

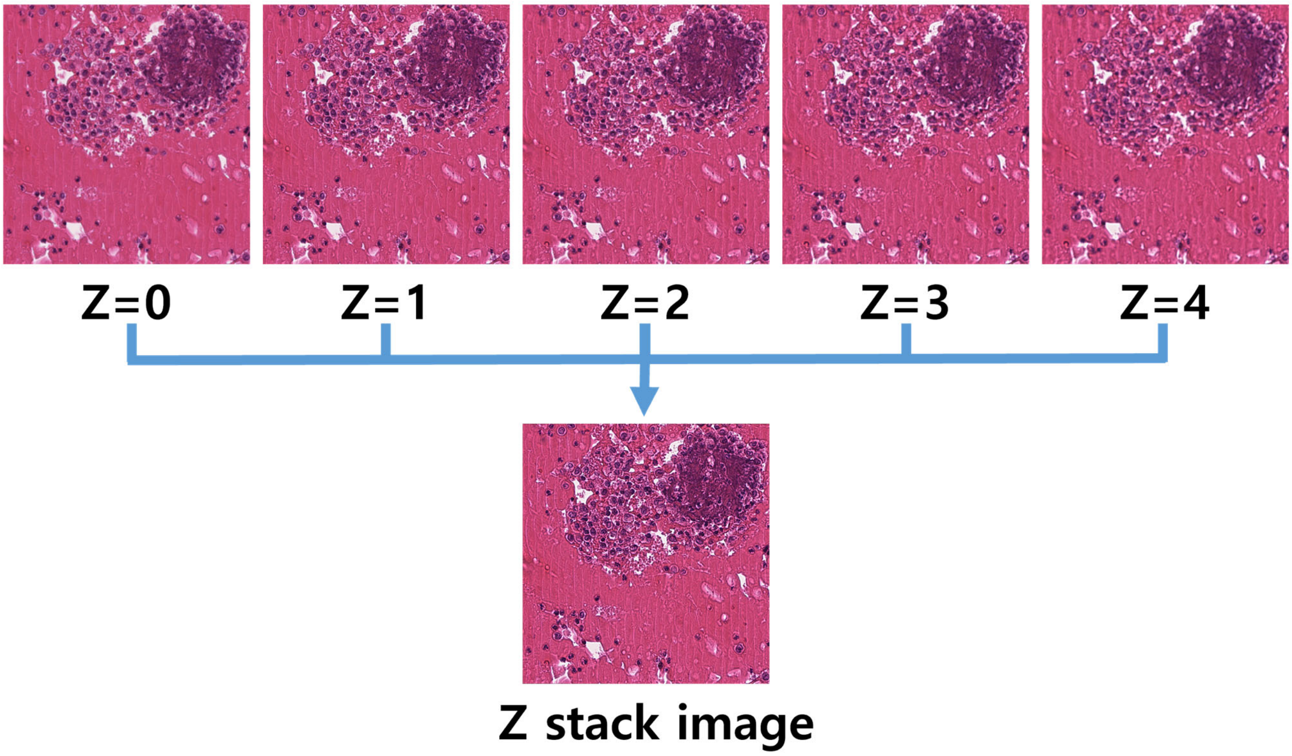

Figure S2. Extended Z-stacking image methods.

A single, high-precision image of cell clusters with different focal planes was obtained by merging five stack images from the conventional slide and three stack images from the liquid-based slide.

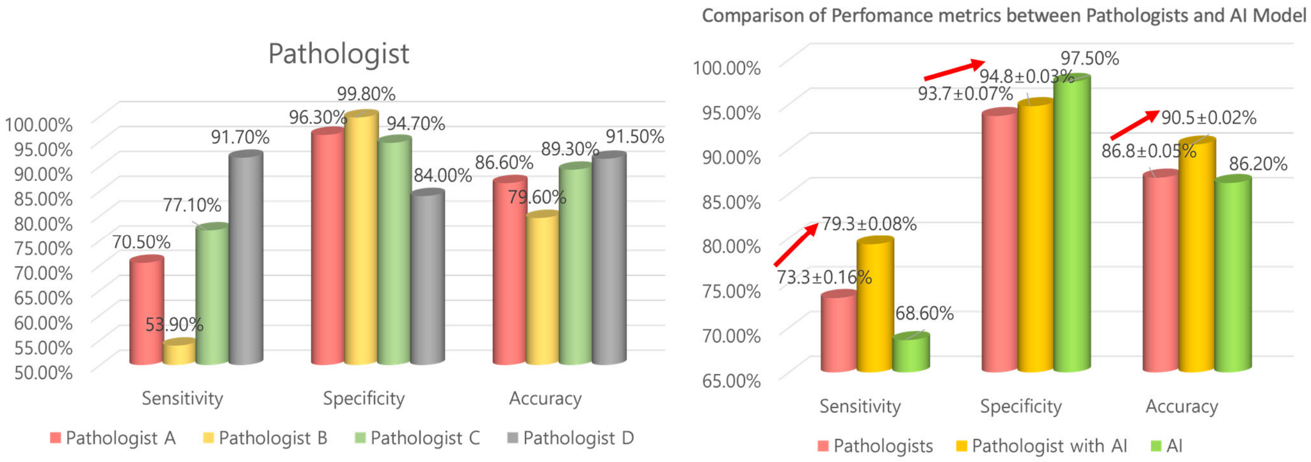

Figure S3. Comparison between pathologists and the AI model.

A total of 1,041 patch images were used to obtain diagnoses by four pathologists, with an average sensitivity of 73.3%, a specificity of 93.7%, and an accuracy of 86.8%. After a re-diagnosis by pathologists based on the AI model, the diagnostic accuracy increased from 86.8% to 90.5%.

Supplement Table:

Table S1. Institutions participated in data collection for this study of the Open AI dataset.

**Hospital**	**Malignant WSI No.**		**Benign WSI No.**		**Total WSI No.**	
	**H&E**	**PAP**	**H&E**	**PAP**	**H&E**	**PAP**
Andong Hospital		1				1
Bucheon Sejong Hospital		1				1
Busan ST. Mary's Hospital		1				1
Catholic University of Korea, College of Medicine, Seoul St. Mary's Hospital		1				1
Catholic University of Korea, College of Medicine, Yeouido St. Mary's Hospital		1				1
Catholic University of Korea, College of Medicine, Uijeongbu St. Mary's Hospital	4	20	15	278	19	298
Gyeongsang National University Hospital				1		1
Good Gang-An Hospital		1				1
Hanyang University Guri Hospital		1				1
Kosin University Gosoel Hospital		1				1
Kangdong Sacred Heart Hospital		1				1
Maryknoll Hospital		1				1
National Cancer Center (NCC)	4*	4*	15*	35*	19*	39*
Saegyaero Hospital		1				1
Samsung Medical Center		1				1
Samsung Changwon Hospital		1				1
Sanggye Paik Hospital		1				1
Serim General Hospital		1				1
St. Mary's Pathology Diagnostic Center				1		1
T&C clinic of pathology				1		1
Yeungnam University Medical Center		1				1
Yonsei University, Gangnam Severance Hospital		1				1
Yonsei University, Severance Hospital	31		120	35	151	35
**Total 24 institutes**	**39**	**41**	**150**	**351**	**189**	**392**

Table S2. Accuracy of PAP and H&E AI algorithms.

**Binary classification**	**PAP**			**H&E**		
**Model**	Accuracy	Sensitivity	Specificity	Accuracy	Sensitivity	Specificity
**Densenet161**	0.7855	0.5553	1.0000	0.9513	0.9140	0.9894
**Dpn**	0.7748	0.5457	1.0000	0.9524	0.9418	0.9632
**Inceptionres-netv2**	0.8544	0.7072	0.9992	0.8969	0.9017	0.8917
**Inceptionv4**	0.8931	0.7845	1.0000	0.9373	0.8863	0.9894
**Mobilenetv2**	0.8745	0.7469	1.0000	**0.9667**	0.9382	0.9957
**Resnet152**	0.8414	0.6801	1.0000	0.9495	0.9038	0.9963
**Resnext**	0.8441	0.6770	1.0000	0.9480	0.9489	0.9879
**Senet154**	0.8697	0.7372	1.0000	0.9498	0.9032	0.9973

**Xception**	**0.8954**	0.7890	1.0000	0.9430	0.8976	0.9894
**Densenet161**	0.7855	0.5553	1.0000	0.9513	0.9140	0.9894
